# Factors associated with insomnia symptoms over three years among premenopausal women with breast cancer

**DOI:** 10.1007/s10549-023-07058-z

**Published:** 2023-08-05

**Authors:** Chloe M. Beverly Hery, Sarah A. Janse, Kimberly J. Van Zee, Elizabeth Z. Naftalis, Electra D. Paskett, Michelle J. Naughton

**Affiliations:** 1 Division of Epidemiology, College of Public Health, The Ohio State University, Columbus, OH 43210 USA; 2 Center for Biostatistics, Department of Biomedical Informatics, The Ohio State University, Columbus, OH 43210 USA; 3 Breast Service, Department of Surgery, Memorial Sloan Kettering Cancer Center, New York, NY 10065 USA; 4 Director of Breast Services, Health Texas Community Health Services Corporate, Dallas, TX 75001 USA; 5 Division of Cancer Prevention and Control, Department of Internal Medicine, College of Medicine, The Ohio State University, Columbus, OH 43210 USA

**Keywords:** Sleep, Insomnia, Breast cancer, Premenopausal, Quality of life, Survivorship

## Abstract

**Purpose:**

We examined longitudinal trends and factors associated with insomnia over 3 years in a cohort of young breast cancer patients.

**Methods:**

Women with stage I–III breast cancer at ≤ 45 years were recruited at five institutions from New York, Texas, and North Carolina, within 8 months of diagnosis (*n* = 836). Participants completed questionnaires every 6 months for 3 years. Linear mixed-effects models were used to examine insomnia over time, using the Women’s Health Initiative Insomnia Rating Scale (WHIIRS). We evaluated the relations of insomnia with demographic (age, race, education, income, employment, marital status), clinical (cancer stage, histologic grade, chemotherapy, radiation, hormone therapy, surgery, tumor size, body mass index, hot flashes), and social/behavioral variables (smoking status, social support, physical activity, depressive symptoms).

**Results:**

At baseline, 57% of participants met or exceeded the cut-off for clinical insomnia (WHIIRS score ≥ 9). Insomnia symptoms were most prevalent at baseline (*p* < 0.0001), but decreased significantly throughout follow-up (*p* < 0.001). However, 42% of participants still experienced insomnia symptoms 3 years after diagnosis. In multivariable models, older age (*p* = 0.02), hot flashes (*p* < 0.0001), and depressive symptoms (*p* < 0.0001) remained significantly associated with insomnia over time.

**Conclusions:**

Insomnia symptoms were most frequent closer to breast cancer diagnosis and treatment, but persisted for some women who were older and those reporting higher hot flashes and depressive symptoms. Survivorship care should include assessing insomnia symptoms, particularly during and immediately after primary treatment. Implementing early interventions for sleep problems may benefit young breast cancer survivors and improve their quality of life.

## Introduction

Over 4 million women in the United States (U.S.) are breast cancer survivors, with an average 5-year survival rate of 91% [1]. Most young survivors will live for many years after their diagnosis, and it is important to ensure high quality of life post-treatment. Although survival rates have improved in young women [2, 3], quality of life may suffer due to the long-term consequences of treatment, psychosocial effects, and early menopause [4–7]. One of the most concerning quality of life issues among cancer survivors is sleep problems [8–11].

Women with breast cancer report some of the highest rates of sleep disturbance, ranging from 59 to 90% [12–15]. Frequently reported problems include poor sleep quality and sleep disturbances [16]. Sleep changes begin during initial treatment with chemotherapy and/or radiation therapy and may persist over the long-term [17, 18], in part, because chemotherapy can disrupt circadian rhythms and increase menopausal symptoms [19, 20]. In a longitudinal study by Savard et al. [21], insomnia rates were highest at the time of breast cancer surgery (69%), and while they observed lower levels 18-months after diagnosis, insomnia was still present in 42% of participants. Another longitudinal study (mean age of 58 years) found fairly consistent insomnia rates over 12 months after cancer diagnosis, which tended to be much higher in prevalence (50%) than the general population (8–10%) [22]. Ongoing sleep problems may contribute to quality of life issues and poorer health status among survivors [10], yet very little has been reported on long-term sleep in breast cancer survivors < 50 years old.

While sleep disturbances are common among all breast cancer survivors [23], younger women face unique additional challenges [11]. Breast cancer at younger ages often is characterized as more aggressive and worse outcomes [24, 25]. These women experience long-term and late effects of cancer treatments, such as treatment-associated fertility issues and amenorrhea [4, 7, 26], in addition to depression and fear of recurrence, more often than older women [27, 28]. Not only do cancer treatments and concerns negatively affect young breast cancer survivors, quality of sleep also influences physical and mental health. High quality sleep is crucial for optimal functioning [29] and is especially important when recovering from cancer. Sleep disruption is a predictor of overall survival in women with breast cancer, with less disrupted sleep being a significant predictor of lower mortality and insufficient sleep being associated with increased mortality [30, 31].

Despite there being a large population of breast cancer survivors, longitudinal studies of sleep in young cancer survivors are limited, but are important in order to provide information on the patterns of sleep changes over time. Most of the current literature on sleep health in breast cancer survivors is summarized across wide age groups, and most breast cancer survivors are over age 50. Therefore, the objective of our study was to examine trends and factors associated with insomnia symptoms over 3 years in a cohort of premenopausal breast cancer survivors.

## Methods

### Study population

We conducted a secondary data analysis of participants (*n* = 836 women) recruited to the Menstrual Cycle Maintenance and Quality of Life After Breast Cancer Study, a multi-center, longitudinal observational study. While eligibility rates and participation rates are not available, most women who were eligible participated in this study. Inclusion criteria for the main study consisted of female patients, ages 18–45 years, diagnosed with stage I–III invasive breast cancer within the previous 8 months [32]. Patients were excluded if they had any prior or concurrent history of any cancer, excluding basal or squamous cell skin carcinoma and stage 0 cervical cancer. Participants were required to have regular menstrual cycles at the time of diagnosis to examine the primary study aims regarding fertility and menopause post-breast cancer treatment. Thus, women who had a previous hysterectomy were ineligible.

Recruitment began in January 1998 and ended in November 2005. Participants were enrolled through five sites: Memorial Sloan-Kettering Cancer Center, New York City, New York; M.D. Anderson Cancer Center, Houston, Texas; University of Texas–Southwestern, Dallas, Texas; Presbyterian Hospital, Dallas, Texas; and Wake Forest University Baptist Medical Center, Winston-Salem, North Carolina. Patients from each clinical center were identified soon after diagnosis using tumor or surgical registries and patient or physician referrals. Patients self-completed baseline forms at study enrollment. Follow-up data collection was completed at 6-month intervals through 36 months post-recruitment. All follow-up data collection was conducted via mail.

Written informed consent was obtained from all participants. This study was approved by the Institutional Review Board of each site and the U.S. Army Medical Research and Materiel Command Human Subjects Committee.

### Measures

#### Insomnia symptoms

The Women’s Health Initiative Insomnia Rating Scale (WHIIRS), a measure of sleep quality and perceived insomnia symptoms over the past 4 weeks, was completed at 6-month intervals from baseline through 3 years post-recruitment: “Did you have trouble falling asleep?”; “Did you wake up several times at night?”; “Did you wake up earlier than you planned to?”; “Did you have trouble getting back to sleep after you woke up too early?”; “Overall, was your typical night’s sleep during the past 4 weeks” Response categories for the first four items ranged from “no, not in the past 4 weeks” (0) to “Yes, 5 or more times a week” (4). The last question responses ranged from “Very sound or restful” (0) to “Very restless” (4). Responses were summed, higher WHIIRS scores (range 0–20) indicate more sleep disruption, with scores ≥ 9 indicative of a clinical diagnosis of insomnia [33, 34].

#### Demographics

We collected demographic information on age at diagnosis, race, marital status, education, total annual household income, and employment status.

#### Medical chart review/clinical factors

Extensive chart reviews were performed at the recruiting institution. Information was obtained on the patients’ height and weight at diagnosis, to calculate body mass index (BMI), cancer stage, tumor size, histologic grade, cancer surgery, receipt of radiation therapy (yes/no), and use of hormone therapy for breast cancer treatment (yes/no). Individual chemotherapy regimens and doses were collected from medical records. Women were also asked to report hot flash symptoms and their intensity during the past month, at each 6-month follow up period.

#### Social/behavioral factors

The following social and behavioral factors were ascertained at 6-month intervals from baseline through 3 years post-recruitment. Physical activity level was assessed with questions on the frequency and duration of walking outside the home and participation in various intensity level activities. The responses were assigned a MET score based on intensity level [35], and we computed MET-h/week. Current smoking status was grouped into current, former, and never smokers. Social support was assessed using the MOS Social Support Survey [36], with higher scores indicating higher support (range 0–100). Depressive symptoms were assessed using the 21-item Beck Depression Inventory [37], for which > 30 indicates severe/extreme depressive symptoms (range 0–63).

### Data analysis

Demographic, clinical, and social/behavioral factors at baseline are presented using counts and percentages for categorical variables and means with standard deviations for continuous variables. We used linear mixed-effects models with random effects at the participant-level to examine insomnia symptoms over the 3-year time period. We first evaluated univariate associations of each covariate with insomnia at each time point. All social/behavioral factors (including depressive symptoms) were measured at the same time as the insomnia assessments. Interactions between each covariate listed above and time were also assessed. The interaction of a covariate and time allowed us to examine whether the relationship between each covariate and insomnia changed with time. Next, we created multivariable models, including significant factors (*p* < 0.1) from univariable analyses. We also included known factors related to sleep, regardless of univariable *p*-values, (race, cancer stage, chemotherapy, social support, physical activity, depressive symptoms), in the multivariable models.

Tests for pairwise differences at each 6-month interval were conducted and adjusted for multiple comparisons using Bonferroni’s method [38]. Normality of residuals were checked for each model. Non-significant interactions (*p* > 0.1) were removed in constructing the final model. All analyses were performed in SAS (v9.4, SAS Institute, Inc., Cary, NC, USA).

## Results

Women in our study were on average age 38 years at diagnosis (range 20–45 years). Retention rates across the 36-month study period were as follows: Baseline: 100% (*n* = 836/836), 6-months: 86.7% (*n* = 725/836), 12-months: 84.1%, (*n* = 703/836), 18-months: 78.1%, (*n* = 653/836), 24-months: 72.8%, (*n* = 609/836), 30-months: 47.7% (*n* = 399/836), and 36-months: 66.3% (*n* = 554/836). Of note, the 30-month survey was not offered to all participants. The majority of participants were non-Hispanic white (89%), had at least a 4-year college degree (68%), and made > $75,000 annually (53%). Most women were diagnosed with stage II cancer (52%), were treated with chemotherapy (88%), and had radiation therapy (69%). Equal proportions of women underwent breast-conserving surgery or mastectomy (50.7% vs. 49.3%). Over a third of the participants were overweight (19.3%) or obese (15%), and 53% reported they were inactive or had low levels of physical activity (Table 1).

**Table 1:** Participant socio-demographic factors, clinical/treatment factors, and health/lifestyle factors at baseline (*N* = 836)

Characteristic	Overall sample (*N* = 836) *n* (%)
*Socio-demographics factors*	
Age at diagnosis, years, mean (SD, range)	38 (SD 4.9, range 20–45)
Age group at diagnosis	
< 30 years	56 (6.7)
30–34.9 years	152 (18.2)
35–39.9 years	249 (29.8)
40–45 years	378 (45.3)
Unknown/missing	1
Race & ethnicity	
White (not Hispanic)	740 (88.5)
Black or African American (not Hispanic)	41 (4.9)
Hispanic	31 (3.7)
Asian or Pacific Islander	23 (2.8)
American Indian or Alaskan Native	1 (0.1)
Marital status	
Married/marriage-like relationship	623 (74.5)
Not married/divorced/widowed	213 (25.5)
Education	
≤ High school graduate	74 (8.9)
Some college or technical school	196 (23.4)
≥ College degree	565 (67.6)
Unknown/missing	1
Average annual household income	
< $35,000	120 (14.6)
$35,000–49,999	105 (12.8)
$50,000–74,999	164 (20.0)
$75,000–100,000	136 (16.6)
> $100,000	296 (36.1)
Unknown/missing	15
Employment status	
Employed full-time	414 (49.6)
Employed part-time	119 (14.2)
Full time homemaker	151 (18.1)
Unemployed/retired/disabled/other	151 (18.1)
Unknown/missing	1
*Clinical/treatment factors*	
Cancer stage	
1	344 (41.3)
2	435 (52.0)
3	56 (6.7)
Unknown/missing	1
Tumor size	
< 2 cm	445 (56.8)
2–5 cm	301 (38.4)
> 5 cm	38 (4.9)
Unknown/missing	52
Histologic grade	
I	57 (8.2)
II	221 (31.8)
III	415 (59.8)
Unknown/missing	143
Surgery	
Lumpectomy only	420 (50.7)
Mastectomy	408 (49.3)
Unknown/missing	8
Chemotherapy	
Yes	737 (88.2)
No	99 (11.8)
Radiation therapy	
Yes	578 (69.3)
No	256 (30.7)
Unknown/missing	2
Hormone therapy for cancer treatment	
Yes	496 (59.6)
No	336 (40.4)
Unknown/missing	4
Hot flashes	
None	508 (60.8)
Mild	159 (19.1)
Moderate	121 (14.5)
Severe	47 (5.6)
Unknown/missing	1
*Social/behavioral factors*	
BMI, (kg/m^2^) mean (SD)	24.5 (5.1)
BMI category	
Underweight (< 18.5 kg/m^2^)	33 (3.9)
Normal (18.5 to 24.9 kg/m^2^)	517 (61.7)
Overweight (25 to 29.9 kg/m^2^)	162 (19.3)
Obese (≥ 30 kg/m^2^)	126 (15.0)
Unknown/missing	2
Smoking status	
Never	488 (58.4)
Former	286 (34.2)
Current	62 (7.4)
Physical activity, MET-h/week, mean (SD)	12.7 (15.6)
Physical activity level^a^	
Inactive (≤ 1.7 MET-h/week )	209 (25.0)
Low (1.8 to 8.3 MET-h/week)	231 (27.6)
Medium (8.4 to 20 MET-h/week )	202 (24.1)
High (> 20 MET-h/week )	194 (23.2)
MOS Social Support score, mean (SD)	82.9 (17.8)
Beck Depression Score^b^, mean (SD; range)	10.3 (SD 7.0; range 0–46)
Depressive symptoms level^b^	
None (0–10)	493 (59.0)
Borderline/mild (11–20)	273 (32.7)
Moderate/severe/extreme (≥ 21)	69 (8.3)
Unknown/missing	1
Insomnia^c^ (WHIIRS Score ≥ 9)	
Yes	471 (56.6)
No	361 (43.4)
Unknown/missing	4

^a^Women were classified into four categories of physical activity level: inactive (≤ 1.7 MET-h/week), low (1.8–8.3 MET-h/week), medium (8.4–20 MET-h/week), and high activity (> 20 MET h/week), where accumulating 150 min/week of moderate-intensity exercise (i.e. the minimum dose of activity recommended by the federal government) (15), as being equivalent to at least 8.4 MET-h/week of exercise [35]

^b^Beck Depression Inventory groups scoring: 1–10 (normal); 11–16 (mild mood disturbance); 17–20 (borderline clinical depressive symptoms); 21–30 (moderate depressive symptoms); 31–40 (Severe depressive symptoms); Over 40 (extreme depressive symptoms) [37]

^c^Insomnia Scoring: The WHIIRS consists of five questions that assess insomnia and sleep quality during the past 4 weeks. Response categories for these items range from 0 (“no, not in the past 4 weeks”) to 4 (“yes, 5 or more times a week”). Higher WHIIRS scores (range 0–20) indicate poorer sleep quality, with scores ≥ 9 indicative of a clinical diagnosis of insomnia [33, 34]

**Fig. 1:**
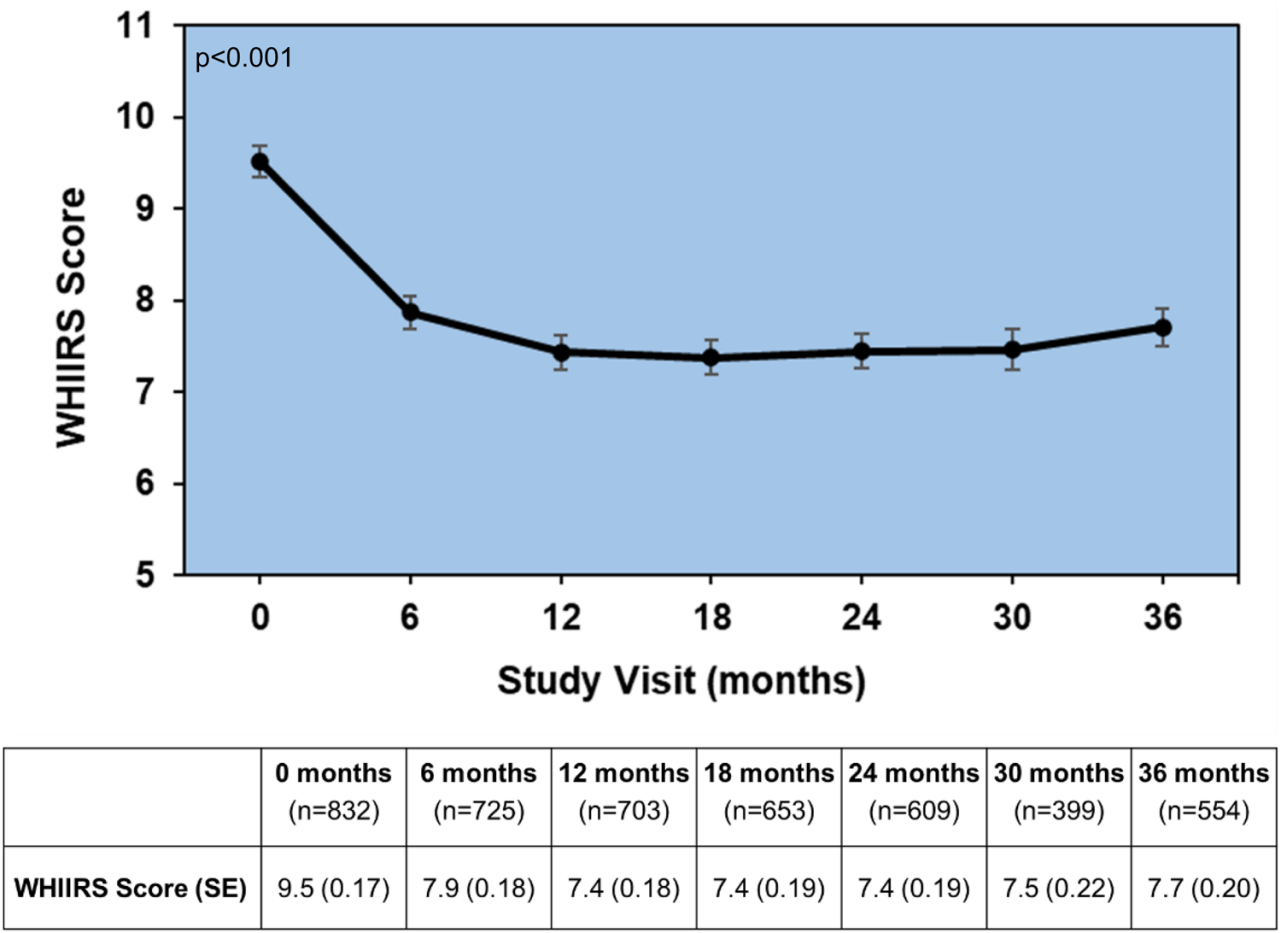
Mean Unadjusted WHIIRS insomnia symptoms’ scores over the 36-month study follow up, with standard error bars. *p* < 0.001—was the significance level of the test for the overall change in sleep scores over time

At baseline, 56.6% of the participants had WHIIRS scores consistent with clinical insomnia (score ≥ 9). The proportion of women with insomnia symptoms decreased to 44% at 6 months and ranged from 37 to 42% across the remaining study period. Baseline insomnia percentages were similar for women who completed the 36-month follow-up compared to women who dropped out (57.0% vs. 55.7%, respectively). The mean WHIIRS score at baseline was 9.5 (Standard Error, SE: 0.17). The score decreased to 7.9 (SE: 0.18) at 6 months. From 12 to 36 months, the average WHIIRS scores stayed stable at 7.4 (SE: 0.18) at 12 months, 7.4 (SE: 0.19) at 18 months, 7.4 (SE: 0.19) at 24 months, 7.5 (SE: 0.22) at 30 months, and 7.7 (SE: 0.20) at 36 months (Fig. 1). Insomnia symptoms decreased significantly over the 3-year period (*p* < 0.001), with baseline scores being significantly worse (more insomnia symptoms) compared to all other assessment points (*p* < 0.0001).

Univariable and multivariable analyses were performed to determine the association between socio-demographic, clinical/treatment, and social-behavioral factors and insomnia symptoms (Table 2). Univariate analyses were performed as a first step. Factors with main effects strongly related to insomnia over time (*p* < 0.1) were older age at diagnosis (*p* = 0.01), lower educational levels (*p* = 0.004), having received chemotherapy (*p* = 0.005), use of hormone therapy (yes/no) (*p* = 0.07), higher BMI (*p* = 0.0007), being a former or current smoker (*p* = 0.0003), having hot flashes (*p* < 0.0001), lower social support (*p* < 0.0001), lower physical activity (*p* < 0.0001), and greater depressive symptoms (*p* < 0.0001). We explored differences by individual chemotherapy regimens, but did not observe any significant findings by regimen type, and thus categorized the variable only as having received chemotherapy (yes/no).

**Table 2:** Univariate and multivariate estimates of factors associated with insomnia symptoms over time

Characteristic	Univariate analysis				Multivariate analysis			
	Estimate^a^	SE	*p*-value	Overall * p*-value (Type 3 fixed effects)	Estimate^a^	SE	*p*-value	Overall * p*-value (Type 3 fixed effects)
Age group at diagnosis				0.01				0.02
< 30 years	− 0.76	0.88	0.38		− 1.43	0.49	0.003	
30–34.9 years	− 0.31	0.55	0.57		− 0.38	0.32	0.24	
35–39.9 years	− 0.92	0.47	0.05		− 0.54	0.27	0.05	
40–45 years	Ref.	Ref.	Ref.		Ref.	Ref.	Ref.	
Race & ethnicity				0.66				0.11
White (not Hispanic)	Ref.	Ref.	Ref.		Ref.	Ref.	Ref.	
Black or African American (not Hispanic)	− 1.23	0.83	0.14		− 0.69	0.58	0.23	
Hispanic	− 1.09	0.95	0.25		− 1.25	0.60	0.04	
Asian or Pacific Islander	− 1.41	1.10	0.20		− 0.67	0.71	0.34	
Education level				0.004				0.68
≤ High school	Ref.	Ref.	Ref.		Ref.	Ref.	Ref.	
Some college	− 0.29	0.68	0.67		0.02	0.46	0.97	
≥ Four-year college degree	− 0.31	0.62	0.62		− 0.22	0.44	0.62	
Average annual household income				0.12				
< $50,000	1.49	0.51	0.003					
$50,000–74,999	1.01	0.55	0.07					
$75,000–100,000	1.08	0.60	0.07					
> $100,000	Ref.	Ref.	Ref.					
Employed				0.94				
Full-time or part-time employment	Ref.	Ref.	Ref.					
Homemaker/retired/other	− 0.13	0.46	0.78					
Marital status				0.52				
Married/marriage-like relationship	Ref.	Ref.	Ref.					
Not married/divorced/widowed	0.50	0.48	0.30					
Cancer stage				0.30				0.56
1	− 0.93	0.92	0.31		0.05	0.52	0.92	
2	− 1.20	0.91	0.19		− 0.21	0.49	0.66	
3	Ref.	Ref.	Ref.		Ref.	Ref.	Ref.	
Histologic grade				0.76				
I	Ref.	Ref.	Ref.					
II	0.59	0.74	0.43					
III	1.37	0.71	0.05					
Chemotherapy				0.005				0.06
No	Ref.	Ref.	Ref.		Ref.	Ref.	Ref.	
Yes	1.58	0.60	0.008		0.72	0.39	0.05	
Radiation therapy				0.89				
No	Ref.	Ref.	Ref.					
Yes	− 0.11	0.43	0.60					
Hormone therapy for cancer treatment				0.07				0.75
No	Ref.	Ref.	Ref.		Ref.	Ref.	Ref.	
Yes	0.16	0.41	0.70		− 0.08	0.24	0.75	
Tumor size				0.66				
< 2 cm	0.002	1.07	0.99					
2–5 cm	0.07	1.09	0.95					
> 5 cm	Ref.	Ref.	Ref.					
Surgery type				0.70				0.62
Lumpectomy only	Ref.	Ref.	Ref.		Ref.	Ref.	Ref.	
Mastectomy	− 0.04	0.40	0.91		0.12	0.24	0.62	
BMI, (kg/m^2^) (continuous)	0.06	0.04	0.12	0.0007	0.02	0.02	0.34	0.34
Smoking status				0.0003				0.33
Never	Ref.	Ref.	Ref.		Ref.	Ref.	Ref.	
Former	1.09	0.43	0.01		0.32	0.25	0.12	
Current	1.52	0.79	0.05		0.46	0.47	0.33	
Hot flashes				< 0.0001				< 0.0001
None	Ref.	Ref.	Ref.		Ref.	Ref.	Ref.	
Mild	0.94	0.37	0.01		1.03	0.15	< 0.0001	
Moderate	2.38	0.43	< 0.0001		2.46	0.18	< 0.0001	
Severe	4.85	0.73	< 0.0001		3.56	0.27	< 0.0001	
Social support	− 0.03	0.008	0.002	< 0.0001	0.005	0.005	0.31	0.31
Physical activity level (MET-h/week)	− 0.02	0.009	0.02	< 0.0001	− 0.008	0.004	0.08	0.08
Beck Depression Score	0.29	0.02	< 0.0001	< 0.0001	0.26	0.01	< 0.0001	< 0.0001

^a^Positively signed estimates are indicative of higher WHIIRS scores which corresponds to more insomnia symptoms/poorer sleep quality. Negatively signed estimates are indicative of lower WHIIRS scores which corresponds to less insomnia symptoms/better sleep quality

Some factors associated with insomnia symptoms were also significant in the interaction with time, including educational level (*p* = 0.03) and receiving chemotherapy (*p* = 0.02). Additionally, race (*p* = 0.05), cancer stage (*p* = 0.02), and surgery type (*p* = 0.02) were significant in the interaction with time, but not in the main effects (not reported in Table 2). In terms of education level, women with less than a college degree had decreased insomnia symptoms scores, but not to the same lower extent as women with higher education. Women at all three cancer stages saw the same decrease in insomnia symptoms scores by 3 years, but women with stage III cancer took longer to see the improvement. Women who received a mastectomy or lumpectomy had similar low insomnia symptoms scores at 36-months. We did not find any significant associations of insomnia symptoms scores with income, employment status, marital status, histologic grade, radiation therapy, or tumor size (all *p*s > 0.1).

**Fig. 2:**
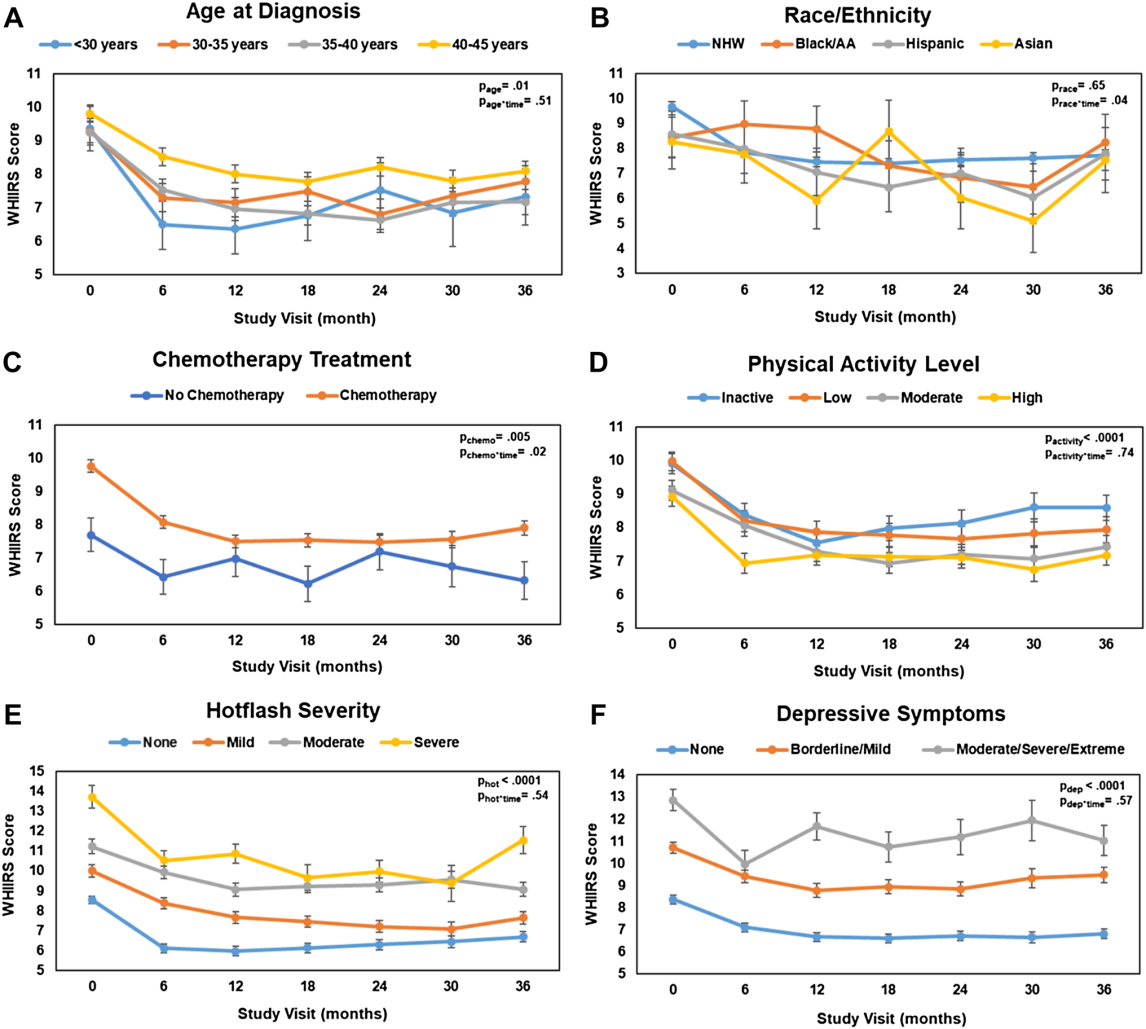
**A**–**F** Selected factors associated with WHIIRS insomnia symptoms scores over the 36-month study follow up (unadjusted plots)

Known factors related to insomnia over time (chemotherapy, physical activity, hot flash severity, and depressive symptoms) and our factors of interest (age and race), were plotted over time to represent the relationship visually. While trends over time were not significantly different across age groups (*p* = 0.51), women who were younger at diagnosis tended to report fewer insomnia symptoms (*p* = 0.01), (Fig. 2A). Non-Hispanic white women saw improvements in insomnia symptoms in the first 6 months that stayed consistent over time (Fig. 2B). Asian-American and Hispanic women also saw a decrease in insomnia symptoms in the first 6 months, but non-Hispanic Black women did not have a decrease in insomnia symptoms until the 18-month time point. Women who did not receive chemotherapy had consistently less insomnia symptoms than women who received chemotherapy (Fig. 2C) and women with the highest level of physical activity had the lowest level of insomnia symptoms (*p* < 0.0001), but this relationship did not vary over time (*p* = 0.74, Fig. 2D). Additionally, those with the most severe hot flashes and the highest levels of depressive symptoms consistently had more insomnia symptoms (*p*s < 0.0001), which was consistent over time (*p*s > 0.54, Fig. 2E and F).

Based on the results of the univariate analysis, we conducted a multivariable analysis including all of the significant factors listed above (*p* < 0.1, Table 2). In the multivariable model, older age at diagnosis (*p* = 0.02), hot flashes (*p* < 0.0001) and higher depressive symptoms scores (*p* < 0.0001) remained significantly associated with insomnia symptoms scores over time. Chemotherapy was borderline significant (*p* = 0.06). Race (*p* = 0.11), education level (*p* = 0.68), cancer stage (*p* = 0.56), hormone therapy for cancer treatment (*p* = 0.75), surgery type (*p* = 0.62), BMI (*p* = 0.34), and smoking status (*p* = 0.33) were no longer significant in the adjusted models. Interestingly, social support (*p* = 0.31) and physical activity (*p* = 0.08), which are often associated with insomnia symptoms and sleep quality, were not significant in the adjusted models, although physical activity level was marginally significant (*p* = 0.08).

## Discussion

Our study assessed insomnia symptoms over 36 months in a sample of premenopausal breast cancer survivors ≤ 45 years old. Insomnia symptoms were highest at baseline, within 8 months of the participants’ breast cancer diagnosis and treatment, but improved in the following years. Insomnia symptoms, however, persisted over the 3-year study period for some young survivors. After adjusting for factors significantly related to insomnia symptom scores in univariate analyses and those known to be related to insomnia symptoms, participants in our study who were older at diagnosis, had hot flashes, and/or had higher depressive symptom scores were significantly more likely to experience persistent insomnia symptoms in our longitudinal analysis.

Disrupted sleep during breast cancer treatment and immediately following is common [17, 18]. In our study, insomnia symptoms were highest (indicating more sleep disruption and worse sleep quality, WHIIRS Score = 9.5) at the baseline visit. Insomnia symptoms decreased over the following 6 months (approximately 1 year after diagnosis, WHIIRS Score = 7.9), then stayed roughly the same at each of the remaining study timepoints from 12 to 36 months.

A key finding in our study is that the proportion of premenopausal women reporting insomnia symptoms was 57% at baseline and 42% at 36 months. This is a substantially high percentage of women experiencing insomnia symptoms 3 years after diagnosis. These findings are consistent with a study by Savard that examined insomnia symptoms over 18 months in cancer patients [21]. They found that 59% of cancer patients (various cancer types) reported insomnia symptoms or insomnia syndrome at baseline, which was before or soon after cancer surgery [21]. They also observed a decrease in insomnia symptoms over time, but noted that insomnia rates remained elevated among some participants, with 36% prevalence at 18 months. A noticeable difference between our study and that of Savard [21] is that the mean age in Savard’s study was older (57 years).

In a more recent study, Fleming et al. [22] reported a similar percentage of breast cancer patients with insomnia around the time of diagnosis (46%). The prevalence of insomnia remained high throughout the 12-month study (50%). Again, the mean age in their population was similar to those in Savard’s (58 years vs 38 years in our study) [22].

Another recent breast cancer study [39] assessed insomnia over 12 months using trajectory modeling (mean age 56 years). Participants were enrolled within 4 months of breast cancer diagnosis, and also used the WHIIRS. In the elevated trajectory group (consisting of women who started out above the WHIIRS cutoff of ≥ 9, but then showed improvement), 56% of women at baseline were above the cutoff and 35.6% remained above the cutoff at 12 months.

Even with these studies of insomnia among breast cancer survivors, little research has examined insomnia symptoms longitudinally in young breast cancer survivors. Most recent publications have included multiple cancer sites or women > 50 years [21, 22, 39]. However, it appears that the prevalence of young breast cancer survivors with insomnia symptoms is similar to that observed among older age groups and those with other cancer types [21, 22, 39]. In our study we found three factors significantly associated with insomnia symptoms over time in young women that remained significant after adjusting for other demographic, clinical, and lifestyle/behavioral variables: age, hot flashes, and depressive symptoms.

Women diagnosed between the ages 40 and 45 years old were found to have worse sleep quality than women diagnosed at < 40 years. This finding persisted even after adjusting for the presence of hot flashes. Hot flashes are a common complaint in breast cancer survivors [40, 41], with as many as 65% of survivors experiencing hot flashes [42]. Hot flashes that occur at night and night sweats are particularly disruptive to sleep and often result in many brief awakenings, and difficulty falling back to sleep [42, 43]. Hot flashes and night sweats are associated with insomnia symptoms, and our findings are consistent with those of others in the cancer survivorship literature [13, 44]. The women in our study who were ≥ 40 years were more likely to have irregular menstrual cycles or become permanently amenorrheic as a result of their cancer treatment. Many of these women also experienced hot flashes. The effect of hot flashes on sleep quality was a graded response, such that the more severe the hot flashes were, the worse sleep that was experienced.

Lastly, higher depressive symptom scores were associated with sleep problems in our study population. In general, young breast cancer survivors tend to report greater depressive symptoms compared to older women with breast cancer [26, 45–47]. A majority of people experiencing depressive symptoms also experience sleep quality complaints (upwards of 90%) [48, 49], and depression is associated with higher insomnia risk in breast cancer patients [41, 47, 50]. Depressive symptoms, and hot flashes, are two key factors associated with poor sleep quality that are frequently reported among breast cancer survivors [47].

We acknowledge several limitations to this research. We used self-reported insomnia and did not have objective sleep data. However, the WHIIRS is a well-validated, widely used self-report measure of insomnia [33, 34]. Participants did not have a measure of sleep collected prior to their diagnosis and enrollment. Therefore, we cannot compare insomnia levels from pre-diagnosis to post-diagnosis. While depressive symptoms were included in our models, we did not consider anxiety which is also important relating to insomnia and should be considered in future studies. Also, while we had information on the severity of hot flashes, we did not have information on the frequency at which these hot flashes occurred, which may influence the amount of sleep disruption observed. A control group consisting of healthy, young women without a cancer diagnosis was also not included in this analysis. However the sleep outcomes were part of symptom analyses to identify what types of sleep interventions were needed in this breast cancer population to improve their quality of life and functioning. Additionally, there is the potential for survival bias in our sample, with only the healthiest participants remaining in the study. However, we did not observe a higher study dropout due to higher cancer stage over the course of this study. Another limitation to note is that we did not have eligibility rates and participation rates of the sample, therefore we cannot comment on the potential for participation bias, yet we do know participation rates were high and most women who were approached and eligible for the study decided to participate. The study population also lacks diversity in terms of race and ethnicity, as unfortunately the exclusion criteria partially contributed to this, with higher rates of hysterectomy in Black/African American populations. In our multivariable models, racial group was ultimately not significant, but this may have been due to lack of adequate statistical power to detect a difference with smaller numbers. However, our study’s strengths include the large sample size, multiple insomnia assessments completed at the same time as relevant demographic, clinical, and behavioral covariables, rigorous longitudinal analysis of the data, and a longer follow up than most prior studies of premenopausal women.

Understanding young breast cancer survivors’ supportive needs after treatment is important for their continued well-being, including sleep quality. Younger women may be more at risk than older patients for persistent symptom concerns and are at a different stage of their lives than most breast cancer patients diagnosed after menopause. Our findings highlight the importance of managing hot flashes and depressive symptoms, which can be extended to improving sleep health. Sleep interventions can be timed with the assistance of longitudinal study findings, such as this one, to include inquiring about and addressing sleep problems during and immediately after cancer treatment. Improving sleep has been shown to increase quality of life and engagement with cancer treatment and follow-up [51], which is especially important for optimal and long-term cancer survivorship for women diagnosed at young ages.

## Data Availability

The datasets generated during and/or analyzed during the current study are not publicly available, because the study was initiated and completed prior to the NIH data sharing guidelines. Data sharing may be available from the corresponding author on reasonable request.
